# A non-specialist depression care pathway for adolescents living with HIV and transitioning into adult care in Peru: a nested, proof of concept pilot study

**DOI:** 10.1017/gmh.2021.16

**Published:** 2021-05-26

**Authors:** Jerome T. Galea, Carmen Contreras, Milagros Wong, Karen Ramos, Valentina Vargas, Hugo Sánchez, Renato A. Errea, Leonid Lecca, Molly F. Franke

**Affiliations:** 1 School of Social Work, University of South Florida, Tampa, USA; 2 College of Public Health, University of South Florida, Tampa, USA; 3 Department of Global Health and Social Medicine, Harvard Medical School, Boston, USA; 4 Socios En Salud, Lima, Peru; 5 Harvard School of Public Health, Boston, USA; 6 Epicentro, Lima, Peru

**Keywords:** Adolescents, depression, integrated care, Peru

## Abstract

**Background:**

Adolescents living with HIV (ALWH) are disproportionally impacted by depression, experiencing worse HIV outcomes. Integrated depression and HIV care may support antiretroviral adherence. This study pilot tested for proof of concept a basic depression care pathway for ALWH to inform depression care integration with HIV services in Peru.

**Methods:**

ALWH were screened for depression with the Patient Health Questionnaire-9 (PHQ-9). Participants with PHQ-9 scores of ⩾10 or suicidal ideation (SI) were eligible for Psychological First Aid (PFA) delivered by non-mental health specialists. Participants with PHQ-9 re-assessments of ⩾20 or SI were referred to specialized services.

**Results:**

Twenty-eight (11 female, 17 male) ALWH aged 15–21 years participated; *n* = 20 (71%) identified as heterosexual. Most (18/28) acquired HIV at birth. Baseline PHQ-9 scores were 0–4, *n* = 3 (11%); 5–9, *n* = 9 (32%); 10–14, *n* = 10 (36%); 15–19, *n* = 4 (14%); and 20–27, *n* = 2 (7%). Eleven participants (40%) reported SI. Among participants with PHQ-9 > 4, 92% (23/25) were not severe. Of the 21 (75%) of participants eligible for PFA, *n* = 9 (32%) accepted at least one session, of which *n* = 3 (33%) were linked to specialized care.

**Conclusions:**

A simple care pathway operationalizing depression screening and non-specialist delivered emotional support is a first step toward integrated depression and HIV care for ALWH.

## Introduction

AIDS is the second leading cause of death among adolescents globally (WHO, 2020), and in 2019 alone, an estimated 34,000 youth aged 10–19 succumbed to the disease (UNAIDS, 2020). Suboptimal adherence to antiretroviral therapy (ART) is the primary culprit of AIDS mortality among people living with HIV. However, relative to children and adults, adolescents living with HIV (ALWH) are the least likely to achieve viral suppression, a precursor to HIV treatment failure (Nachega *et al*., 2009; Adejumo *et al*., 2015). Although many factors negatively affect ART adherence, depression both disproportionally affects ALWH compared to other age groups (Mellins *et al*., 2009; Elkington *et al*., 2011; Benton *et al*., 2019) and is associated with worse HIV treatment outcomes (Murphy *et al*., 2001; Naar-King *et al*., 2006; Agwu and Fairlie, 2013).

Left untreated, ALWH with depression can face mounting problems as they approach adulthood; these include poorer quality of life, more rapid progression of HIV and higher mortality rates (Haines *et al*., 2019). Moreover, untreated depression can complicate the transition from pediatric to adult HIV care, during which ALWH already face reduced retention in care, ART adherence, CD4 cell counts and HIV viral load suppression (Agwu and Fairlie, 2013; Adejumo *et al*., 2015).

Accordingly, increasing research demonstrates the benefit of treating comorbid depression and HIV (Sikkema *et al*., 2015; Van Luenen *et al*., 2018), especially among adolescents (Vreeman *et al*., 2017). Increasingly prominent are calls for *integrated* care models (i.e. care pathways) that treat both HIV and depression to achieve improved outcomes for both morbidities (Chibanda, 2017; Echenique *et al*., 2019; Remien *et al*., 2019). Integrating mental health services into common priority health care platforms, including HIV, is part of a larger movement to increase access to mental health services for all people (Patel *et al*., 2013). However, for youth, the literature on mental health care pathways is especially scant except for serious mental illnesses (Macdonald *et al*., 2018). For ALWH, given the importance of a successful transition to adult HIV care on long-term health outcomes, emphasis has been on the development of comprehensive transition interventions that not only directly address ART adherence but psychosocial needs, as well (Machado *et al*., 2010; Righetti *et al*., 2015; Westling *et al*., 2016).

Despite the role that depression plays in ART adherence for all people living with HIV, standardized depression screening and care linkage is not part of the Peruvian National Guidelines for HIV prevention and treatment (MINSA, 2020). The primary objective of the current study was to pilot test for proof of concept a basic depression care pathway for ALWH to inform future depression care integration with HIV services in Peru.

## Methods

### Participants and procedures

The current study was conducted at the Peruvian branch of the international nonprofit organization Partners In Health (locally, Socios En Salud or SES) among ALWH participating in the research intervention ‘PASEO’ to facilitate transition to adult HIV care. SES’ mission is to provide a preferential option for the poor in health care, concentrating services in districts of Lima with high levels of poverty, unemployment, and low access to health care. PASEO participants were between 15 and 21 years of age, living with HIV, enrolled in HIV care at a public clinic, receiving or eligible to receive ART, and transitioning to adult care. Using purposive sampling, we recruited a diverse sample of ALWH for PASEO, including males and females who had acquired HIV recently or at birth/early childhood. Peruvian Ministry of Health providers at high-burden public sector clinics referred adolescents meeting the inclusion criteria to the PASEO study team. Participants ⩾18 years of age provided informed consent in their native language, Spanish, whereas participants <18 provided assent with parental/guardian consent. The PASEO protocol, informed assent/consent, and related materials were reviewed and approved by human ethics boards in Peru and the USA.

The PASEO intervention comprised of community-based activities directly targeting retention in care (i.e. health system navigation support; home visits to assess adherence and barriers to care; ART directly observed therapy) and psychosocial well-being (social support delivered through twice-monthly support groups and interactions with lay- and entry-level health workers; education sessions). The social support groups, led by unlicensed, bachelors-level personnel with degrees in psychology, were included in the PASEO intervention because of their impact on improving mental health and HIV-related treatment outcomes (Funck-Brentano *et al*., 2005; Walstrom *et al*., 2013; Galea *et al*., 2018) but were not intended nor designed to treat depression.

### The depression care pathway

**Fig. 1.**
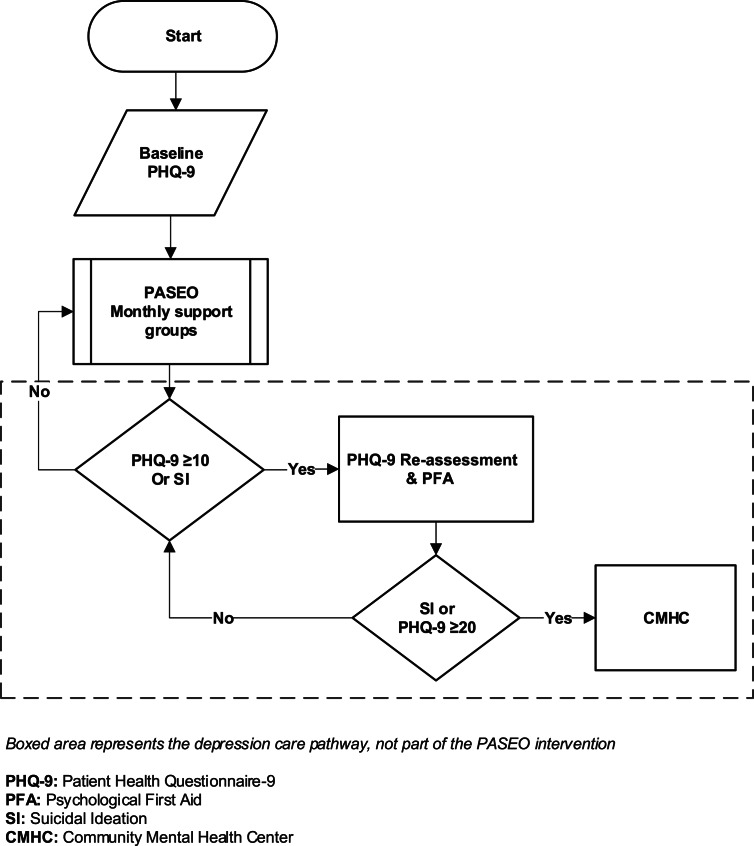
Depression care pathway for adolescents living with HIV (ALWH) participating in the PASEO study.

We developed a depression care pathway that was external to but articulated with PASEO to identify participants with depressive symptoms and provide additional screening, enhanced non-specialist support using Psychological First Aid (PFA), and linkage to free, specialized mental health services provided by the Peruvian Ministry of Health (Fig. 1).

At the beginning of PASEO, baseline data were collected during the first 3 months of study participation, including self-administered, tablet-based depressive symptom screening using the validated Peruvian version of the Patient Health Questionnaire (PHQ-9) (Calderon *et al*., 2012). Participants scoring ⩾10 on the PHQ-9 or with suicidal ideation (SI) were eligible for SES’ in-house mental health program staffed by unlicensed, bachelors-level psychologists (persons trained in psychology with general mental health training). Staff re-assessed depressive symptoms by interviewing the participant to understand their current circumstances, experience, history, and previous treatment of depressive symptoms, and by reapplication of the PHQ-9, after which PFA was provided. PFA is a World Health Organization (WHO)-disseminated intervention designed to provide front-line social and psychological support for people in distress, especially in low- and middle-income countries (LMICs), and can be delivered by laypersons and other non-mental health specialist personnel (WHO, 2011). PFA was selected because it is highly adaptable and permitted staff to focus on immediate emotional support and identification of risk factors for mental health conditions such as SI and violence (e.g. domestic, sexual, gender, psychological, and physical).

After PFA, participants with a PHQ-9 re-assessment score of ⩾20 or SI/risk of self-harm were linked to specialized care at a community mental health center able to meet complex mental health needs beyond which PFA could address. ALWH scoring ⩾10 and ⩽19 on the PHQ-9 re-assessment received additional PFA sessions per Fig. 1. ALWH experiencing economic and/or housing insecurity were referred to SES’ Social Protection program, which provided direct support (e.g. food vouchers) and linkage to other organizations (e.g. group homes for ALWH).

### Measures

Participant-level data included: descriptive (age, sex, gender identity, sexual orientation, and HIV acquisition route/timing); social determinants of health (housing stability and family support); and depressive symptoms (PHQ-9). Depressive symptom severity was computed by summing the overall PHQ-9 score (range 0–27) and reported following the standard cut-offs: 0–4 none/minimal; 5–9 mild; 10–14 moderate; 15–19 moderately-severe; and 20–27 severe (Kroenke *et al*., 2001). SI was captured by PHQ-9 item 9 (How often have you been bothered by the following over the past 2 weeks: Thoughts that you would be better off dead, or thoughts of hurting yourself in some way?) Furthermore, we recorded the number of participants receiving each component of the Depression Care Pathway.

## Results

Between October 2019 and January 2020, PASEO administered the PHQ-9 to 28 ALWH, comprised of 11 females and 17 males, with a mean age of 18.9 years (range 15–21). Most (64%) study participants acquired HIV at birth, and 71% identified as heterosexual (Table 1).

**Table 1.** Characteristics of Peruvian ALWH participating in PASEO with PHQ-9 scores (*N* = 28)

Characteristics	Total *N* = 28, *N* (%)
Age	
15–17	6 (21)
18–21	22 (79)
Sex assigned at birth	
Female	11 (39)
Male	17 (61)
Gender identity	
Woman	11 (39)
Transgender woman	1 (4)
Man	16 (57)
Sexual orientation	
Heterosexual	20 (71)
Homosexual	8 (29)
HIV acquisition	
Vertical/early childhood	18 (64)
Recent	10 (36)
Housing stability^a^	
Stable	12 (43)
Unstable	16 (57)
Family support^b^	
Yes	18 (64)
No	10 (36)

a Stable housing: participant lived in a dwelling (rented or owned) by a family member or other caregiver.

b Family support: participant had at least one family member that provided support (material, emotional).

### Baseline depressive symptoms: frequency and distribution of severity

**Fig. 2.**
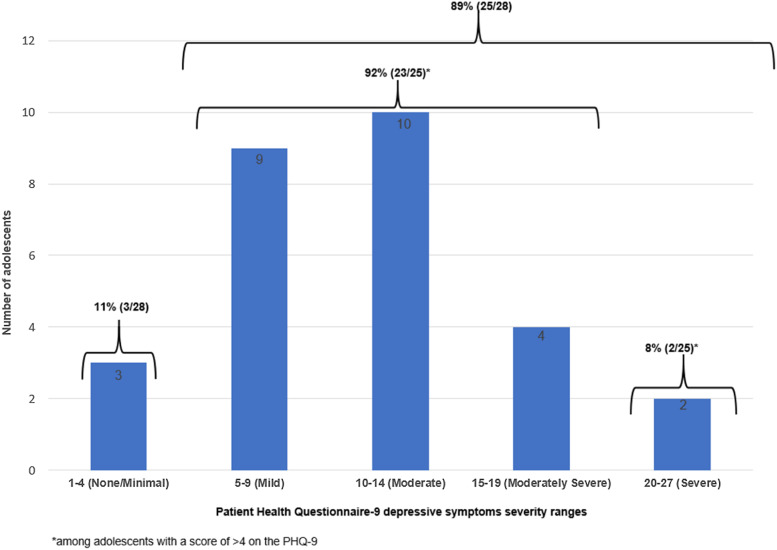
Baseline depressive symptom frequency and severity by PHQ-9 cut-offs.

Frequency of depressive symptoms at baseline was: PHQ-9 = 0–4 (none/minimal), *n* = 3 (11%); PHQ-9 = 5–9 (mild), *n* = 9 (32%); PHQ-9 = 10–14 (moderate), *n* = 10 (36%); PHQ-9 = 15–19 (moderately severe), *n* = 4 (14%); and PHQ-9 = 20–27 (severe), *n* = 2 (7%). Eleven (40%) participants endorsed having suicidal thoughts more than half of the days in the preceding 2 weeks (PHQ-9 item 9). Among participants with a baseline PHQ-9 score >4 (*n* = 25, 89%), 92% (23/25) clustered in the mild- to moderately severe range (Fig. 2).

### Distribution of participants along the depression care pathway

Twenty-one (75%) participants were eligible to enter the depression care pathway, comprising ALWH with a PHQ-9 score of ⩾10 or any SI. Among these *n* = 21 participants, 9 (43%) accepted of which six were re-assessed with the PHQ-9 resulting in *n* = 2 (33%) with PHQ-9 = 5–9 and *n* = 4 (67%) with PHQ-9 = 10–14. Of the three not re-assessed, *n* = 1 was due to staff error but reported SI at baseline, and *n* = 2 were actively reporting SI, making PHQ-9 re-assessment superfluous. All nine participants received at least one session of PFA (range 1–5 sessions). Finally, *n* = 3 (33%) were linked to specialized mental health services (all with SI), and *n* = 6 (67%) egressed the care pathway because their PHQ-9 scores were ⩽20 and they were not reporting SI.

## Discussion

We pilot-tested a depression care pathway for ALWH in parallel with a community-based research study supporting ALWH transitioning from pediatric to adult care, finding that while depressive symptoms were common among study participants, most did not require specialized mental health services. This study demonstrates both the need for and preliminary proof of concept of a simple depression care pathway comprised of existing tools (PHQ-9, PFA), which could eventually be integrated into HIV care services. Although small, our study is also the first to report depressive symptomology among a diverse sample of Peruvian ALWH. Our data complement findings from previous studies of depression among adult Peruvian populations living with HIV (Ferro *et al*., 2015; Maldonado Ruiz *et al*., 2015; Defechereux *et al*., 2016), in which similarly high rates of depression were found.

Despite the high prevalence of depressive symptoms, our finding that near 90% were not severe, according to the PHQ-9, is especially relevant in the context of integrated HIV and depression care service models. Because most of the ALWH with depressive symptoms in our study did not require linkage to specialized care, in theory, the non-specialist PFA provided by SES could be delivered by similar non-specialist personnel within the HIV care delivery system as a first step toward integrated HIV care. This finding is important for two reasons. First, in the larger context of mental health service access in general, there is a global shortage of mental health professionals to deliver care, especially in LMICs where >90% of people with HIV live (UNAIDS, 2018). Thus, integrated HIV and depression care pathways relying on specialized therapies delivered by mental health professionals are unlikely to achieve increased rates of depression care for ALWH, particularly in LMICs, which lack specialized personnel. Second, non-specialists already provide a critical role in the HIV treatment cascade worldwide, including HIV adherence counseling (Bemelmans *et al*., 2016). These non-specialists constitute a ready workforce that could minimally provide depression screening and basic counseling like PFA along with delivering HIV care.

In Peru, non- mental health specialists delivering mental health services are part of a larger national strategy to increase access to care for all people (Toyama *et al*., 2017), and non-specialist depression interventions have been successfully implemented outside of the HIV-service setting (Eappen *et al*., 2018; Scorza *et al*., 2018). In the future, basic depression care pathways like ours could be expanded beyond screening and brief supportive care to include existing, evidence-based depression interventions, such as the WHO's Mental Health Gap Programme (WHO, 2010) delivered by non-specialists. This approach could be of special interest to the >90 countries that already use these ‘low-intensity’ WHO psychological interventions (Keynejad *et al*., 2018) as a pragmatic way to expand depression care services to vulnerable populations such as ALWH (Galea *et al*., 2020). In Peru, it is also essential to note that laypersons delivering mental health services do so in coordination with a growing network of professionally staffed community mental health centers – to date, there are 155 nationwide, two of which are located in SES's catchment area (MINSA, 2020) – to provide acute mental health services. These centers exist with the express purpose of providing mental health support – including home visits – which should allay concerns regarding care availability.

Because this nested proof of concept study was not designed as a formal implementation study, future research should assess the feasibility and acceptability of a depression care pathway using standardized frameworks and measures, which include the views of ALWH, their HIV care providers, and other stakeholders. Further investigation should also include the impact of PFA *v.* other existing low-intensity psychological interventions on depression and HIV care outcomes. We note that less than half (9/21) of the participants in our study accepted the first step in the care pathway, and one participant was not re-assessed for depression due to staff error. Understanding reasons for non-acceptance of depression care and supporting staff fidelity of the care pathway are opportunities to strengthen future iterations of integrated care pathways.

For example, if a barrier to ALWH accepting care were related to the stigma associated with speaking to another person, then self-help options could be included in the care pathway as an alternative care option. To ensure fidelity to the care pathway, increased structure (e.g. a checklist that explicitly tracks where ALWH are along the pathway and the required evaluations needed to progress subsequent steps) should be considered.

## Conclusions

ALWH are disproportionally affected by comorbid depression, which adversely affects their HIV treatment outcomes due to lower ART adherence, especially during the transition from pediatric to adult care. Depression care pathways that operationalize depression screening and provide basic non-specialist delivered emotional support and referral to specialized care represent a first step toward future integrated care models.

## References

[ref1] Adejumo OA, Malee KM, Ryscavage P, Hunter SJ, and Taiwo BO (2015) Contemporary issues on the epidemiology and antiretroviral adherence of HIV-infected adolescents in sub-Saharan Africa: a narrative review. Journal of the International AIDS Society 18, 20049. 26385853 10.7448/IAS.18.1.20049 PMC4575412

[ref2] Agwu AL, and Fairlie L (2013) Antiretroviral treatment, management challenges and outcomes in perinatally HIV-infected adolescents. Journal of the International AIDS Society 16, 18579. 23782477 10.7448/IAS.16.1.18579 PMC3687074

[ref3] Bemelmans M, Baert S, Negussie E, Bygrave H, Biot M, Jamet C, Ellman T, Banda A, Van Den Akker T, and Ford N (2016) Sustaining the future of HIV counselling to reach 90-90-90: a regional country analysis. Journal of the International AIDS Society 19, 20751. 27189531 10.7448/IAS.19.1.20751 PMC4870383

[ref4] Benton TD, Kee Ng WY, Leung D, Canetti A, and Karnik N (2019) Depression among youth living with HIV/AIDS. Child and Adolescent Psychiatric Clinics of North America 28, 447–459. 31076119 10.1016/j.chc.2019.02.014

[ref5] Calderon M, Galvez-Buccollini JA, Cueva G, Ordonez C, Bromley C, and Fiestas F (2012) Validation of the Peruvian version of the PHQ-9 for diagnosing depression. Revista Peruana de Medicina Experimental y Salud Pública 29, 578–579. 23338650 10.1590/s1726-46342012000400027

[ref6] Chibanda D (2017) Depression and HIV: integrated care towards 90-90-90. International Health 9, 77–79. 28115469 10.1093/inthealth/ihw058 PMC5881265

[ref7] Defechereux PA, Mehrotra M, Liu AY, Mcmahan VM, Glidden DV, Mayer KH, Vargas L, Amico KR, Chodacki P, Fernandez T, Avelino-Silva VI, Burns D, Grant RM, and Iprex Study T (2016) Depression and oral FTC/TDF pre-exposure prophylaxis (PrEP) among men and transgender women who have sex with men (MSM/TGW). AIDS and Behavior 20, 1478–1488. 26078115 10.1007/s10461-015-1082-2 PMC4903104

[ref8] Eappen B, Aguilar M, Ramos K, Contreras C, Prom MC, Scorza P, Gelaye B, Rondon M, Raviola G, and Galea JT (2018) Preparing to launch the ‘thinking healthy programme’ perinatal depression intervention in Urban Lima, Peru: experiences from the field. Global Mental Health 5, e41. 30637114 10.1017/gmh.2018.32 PMC6315282

[ref9] Echenique M, Musselman D, Avellaneda VB, Illa L, Rodriguez AE, Wawrzyniak A, and Kolber MA (2019) Integrated mental health and HIV care in a majority minority clinic. Personalized Medicine in Psychiatry 13–14, 1–5.

[ref10] Elkington KS, Robbins RN, Bauermeister JA, Abrams EJ, Mckay M, and Mellins CA (2011) Mental health in youth infected with and affected by HIV: the role of caregiver HIV. Journal of Pediatric Psychology 36, 360–373. 20947561 10.1093/jpepsy/jsq094 PMC3062287

[ref11] Ferro EG, Weikum D, Vagenas P, Copenhaver MM, Gonzales P, Peinado J, Cabello R, Lama JR, Sanchez J, and Altice FL (2015) Alcohol use disorders negatively influence antiretroviral medication adherence among men who have sex with men in Peru. AIDS Care 27, 93–104. 25277252 10.1080/09540121.2014.963013 PMC4221495

[ref12] Funck-Brentano I, Dalban C, Veber F, Quartier P, Hefez S, Costagliola D, and Blanche S (2005) Evaluation of a peer support group therapy for HIV-infected adolescents. AIDS (London, England) 19, 1501–1508. 10.1097/01.aids.0000183124.86335.0a 16135904

[ref13] Galea JT, Wong M, Munoz M, Valle E, Leon SR, Diaz Perez D, Kolevic L, and Franke M (2018) Barriers and facilitators to antiretroviral therapy adherence among Peruvian adolescents living with HIV: a qualitative study. PLoS One 13, e0192791. 29447226 10.1371/journal.pone.0192791 PMC5813958

[ref14] Galea JT, Marhefka S, Cyrus E, Contreras C, and Brown B (2020) Novel approach to scale integrated depression and HIV care. The Lancet HIV 7, e458–e459. 32059784 10.1016/S2352-3018(20)30025-4 PMC8325020

[ref15] Haines C, Loades ME, Coetzee BJ, and Higson-Sweeney N (2019) Which HIV-infected youth are at risk of developing depression and what treatments help? A systematic review focusing on Southern Africa. International Journal of Adolescent Medicine and Health. doi: 10.1515/ijamh-2019-0037, Epub ahead of print. 31393831

[ref16] Keynejad R, Dua T, Barbui C, and Thornicroft G (2018) WHO Mental Health Gap Action Programme (mhGAP) intervention guide: a systematic review of evidence from low and middle-income countries. Evidence-Based Mental Health 21, 30–34. 28903977 10.1136/eb-2017-102750 PMC10283403

[ref17] Kroenke K, Spitzer RL, and Williams JB (2001) The PHQ-9: validity of a brief depression severity measure. Journal of General Internal Medicine 16, 606–613. 11556941 10.1046/j.1525-1497.2001.016009606.x PMC1495268

[ref18] Macdonald K, Fainman-Adelman N, Anderson KK, and Iyer SN (2018) Pathways to mental health services for young people: a systematic review. Social Psychiatry and Psychiatric Epidemiology 53, 1005–1038. 30136192 10.1007/s00127-018-1578-y PMC6182505

[ref19] Machado DM, Succi RC, and Turato ER (2010) Transitioning adolescents living with HIV/AIDS to adult-oriented health care: an emerging challenge. Jornal de Pediatria (Rio J) 86, 465–472. 10.2223/JPED.2048 21140043

[ref20] Maldonado Ruiz H, Peña Olano RF, and Tomateo Torvisco JD (2015) Frecuencia de episodio depresivo mayor y factores relacionados en pacientes infectados por el virus de la inmunodeficiencia humana en tratamiento antirretroviral de gran actividad (TARGA) en un hospital público de Lima. Revista de neuro-psiquiatria 78, 3–13.

[ref21] Mellins CA, Brackis-Cott E, Leu CS, Elkington KS, Dolezal C, Wiznia A, Mckay M, Bamji M, and Abrams EJ (2009) Rates and types of psychiatric disorders in perinatally human immunodeficiency virus-infected youth and seroreverters. Journal of Child Psychology and Psychiatry, and Allied Disciplines 50, 1131–1138. 10.1111/j.1469-7610.2009.02069.x PMC2775808 19298479

[ref22] MINSA (2020) *Te cuido*, *nos cuidamos: por una conviviencia saludable* [Online]. Available at http://www.minsa.gob.pe/salud-mental/.

[ref23] MINSA (2020) Norma Técnica de Salud de Atención Integral del Adulto con Infección por el Virus de la Inmunodeficiencia Humana (VIH). Lima, Peru: Ministerio de Salud del Peru.

[ref24] Murphy DA, Wilson CM, Durako SJ, Muenz LR, and Belzer M (2001) Antiretroviral medication adherence among the REACH HIV-infected adolescent cohort in the USA. AIDS Care 13, 27–40. 11177463 10.1080/09540120020018161

[ref25] Naar-King S, Templin T, Wright K, Frey M, Parsons JT, and Lam P (2006) Psychosocial factors and medication adherence in HIV-positive youth. AIDS Patient Care and STDs 20, 44–47. 16426155 10.1089/apc.2006.20.44

[ref26] Nachega JB, Hislop M, Nguyen H, Dowdy DW, Chaisson RE, Regensberg L, Cotton M, and Maartens G (2009) Antiretroviral therapy adherence, virologic and immunologic outcomes in adolescents compared with adults in Southern Africa. Journal of Acquired Immune Deficiency Syndromes (1999) 51, 65–71. 19282780 10.1097/QAI.0b013e318199072e PMC2674125

[ref27] Patel V, Belkin GS, Chochalingam A, Cooper J, Saxena S, and Unutzer J (2013) Grand challenges. Integrating mental health services into priority health care platforms. PLoS Medicine 10(5), e1001448. doi: 10.1371/journal.pmed.1001448. 23737736 PMC3666874

[ref28] Remien RH, Stirratt MJ, Nguyen N, Robbins RN, Pala AN, and Mellins CA (2019) Mental health and HIV/AIDS: the need for an integrated response. AIDS (London, England) 33, 1411–1420. 10.1097/QAD.0000000000002227 PMC6635049 30950883

[ref29] Righetti A, Prinapori R, Nulvesu L, Fornoni L, Viscoli C, and Di Biagio A (2015) Transitioning HIV-infected children and adolescents into adult care: an Italian real-life experience. Journal of the Association of Nurses in AIDS Care 26, 652–659. 10.1016/j.jana.2015.05.003 26116060

[ref30] Scorza P, Cutipe Y, Mendoza M, Arellano C, Galea JT, and Wainberg ML (2018) Lessons from rural Peru in integrating mental health into primary care. Psychiatric Services 70, 82–84. 30332927 10.1176/appi.ps.201800079 PMC6408283

[ref31] Sikkema KJ, Dennis AC, Watt MH, Choi KW, Yemeke TT, and Joska JA (2015) Improving mental health among people living with HIV: a review of intervention trials in low- and middle-income countries. Global Mental Health 2, e19. 26435843 10.1017/gmh.2015.17 PMC4589870

[ref32] Toyama M, Castillo H, Galea JT, Brandt LR, Mendoza M, Herrera V, Mitrani M, Cutipe Y, Cavero V, Diez-Canseco F, and Miranda JJ (2017) Peruvian mental health reform: a framework for scaling-up mental health services. International Journal of Health Policy and Management 6, 501–508. 28949462 10.15171/ijhpm.2017.07 PMC5582436

[ref33] UNAIDS (2018) *The Global HIV/AIDS Epidemic* [Online]. U.S. Department of Health & Human Services and supported by the Secretary's Minority AIDS Initiative Fund (SMAIF). [Online]. Available at https://www.hiv.gov/hiv-basics/overview/data-and-trends/global-statistics.

[ref34] UNAIDS (2020) *UNAIDS HIV data and estimates* [Online]. Geneva: World Health Organization. [Online]. Available at https://www.unaids.org/en/dataanalysis/knowyourresponse/HIVdata_estimates.

[ref35] Van Luenen S, Garnefski N, Spinhoven P, Spaan P, Dusseldorp E, and Kraaij V (2018) The benefits of psychosocial interventions for mental health in people living with HIV: a systematic review and meta-analysis. AIDS and Behavior 22, 9–42. 28361453 10.1007/s10461-017-1757-y PMC5758656

[ref36] Vreeman RC, Mccoy BM, and Lee S (2017) Mental health challenges among adolescents living with HIV. Journal of the International AIDS Society 20, 21497. 28530045 10.7448/IAS.20.4.21497 PMC5577712

[ref37] Walstrom P, Operario D, Zlotnick C, Mutimura E, Benekigeri C, and Cohen MH (2013) 'I think my future will be better than my past': examining support group influence on the mental health of HIV-infected Rwandan women. Global Public Health 8, 90–105. 22812728 10.1080/17441692.2012.699539 PMC5576858

[ref38] Westling K, Navér L, Vesterbacka J, and Belfrage E (2016) Transition of HIV-infected youths from paediatric to adult care, a Swedish single-centre experience. Infectious Diseases 48, 449–452. 26950534 10.3109/23744235.2016.1143964

[ref39] WHO (2010) WHO Mental Health Gap Action Programme (mhGAP). Geneva: WHO.

[ref40] WHO (2011) Psychological First aid: Guide for Field Workers. Geneva: World Health Organization.

[ref41] WHO (2020) Health for the World's Adolescents: A Second Chance in the Second Decade [Online]. Geneva: World Health Organization. Available at https://apps.who.int/adolescent/second-decade/

